# Mendelian randomization analysis identified potential genes pleiotropically associated with gout

**DOI:** 10.3389/fgene.2024.1426860

**Published:** 2024-08-05

**Authors:** Yu Wang, Jiahao Chen, Hang Yao, Yuxin Li, Xiaogang Xu, Delin Zhang

**Affiliations:** ^1^ Graduate School of Jiangxi University of Traditional Chinese Medicine, Nanchang, China; ^2^ School of Basic Medical Sciences, Zhejiang Chinese Medical University, Hangzhou, China; ^3^ School of Traditional Chinese Medicine, Binzhou Medical University, Yantai, China

**Keywords:** gout, expression quantitative trait loci, pleotropic association, summary data-based mendelian randomization, genome-wide association study

## Abstract

**Background:**

This study aims to prioritize genes potentially involved in multifactorial or causal relationships with gout.

**Methods:**

Using the Summary Data-based Mendelian Randomization (SMR) approach, this research analyzed expression quantitative trait loci (eQTL) data from blood and renal tissues and genome-wide association study (GWAS) data related to gout. It sought to identify genetic loci potentially involved in gout. Heterogeneity testing was conducted with the HEIDI test, and results were adjusted for the False Discovery Rate (FDR). Blood cis-eQTL data were sourced from the eQTLGen Consortium’s summary-level data, and renal tissue data came from the V8 release of the GTEx eQTL summary data. Gout GWAS data was sourced from the FinnGen Documentation of the R10 release.

**Result:**

SMR analysis identified 14 gene probes in the eQTLGen blood summary-level data significantly associated with gout. The top five ranked genes are: ENSG00000169231 (labeled THBS3, P_SMR_ = 4.16 × 10^−13^), ENSG00000231064 (labeled THBS3-AS1, P_SMR_ = 1.88 × 10^−8^), ENSG00000163463 (labeled KRTCAP2, P_SMR_ = 3.88 × 10^−6^), ENSG00000172977 (labeled KAT5, P_SMR_ = 1.70 × 10^−5^), and ENSG00000161395 (labeled PGAP3, P_SMR_ = 3.24 × 10^−5^). Notably, increased expression of KRTCAP2 and PGAP3 is associated with an increased risk of gout, whereas increased expression of THBS3, THBS3-AS1, and KAT5 is associated with a reduced gout risk. No significant gene associations with gout were observed in renal tissue, likely due to the limited sample size of kidney tissue.

**Conclusion:**

Our findings have highlighted several genes potentially involved in the pathogenesis of gout. These results offer valuable insights into the mechanisms of gout and identify potential therapeutic targets for its treatment.

## 1 Introduction

Gout is commonly diagnosed as inflammatory arthritis characterized by hyperuricemia and the deposition of monosodium urate (MSU) crystals, causing intense pain and joint damage, often leading to deformity (Dalbeth et al., 2019). As a multifactorial metabolic disorder, the primary risk factors for gout include hyperuricemia, genetic predispositions, and dietary influences (Kuo et al., 2015). Research shows that gout’s pathogenesis mainly results from abnormal uric acid metabolism—due to either reduced excretion or increased production—which precipitates MSU crystal deposits in joints, triggering inflammation (Yang et al., 2024).

Studies reveal that gout’s global prevalence ranges from 1% to 4%, with an incidence rate of 0.1%–0.3% (Singh and Gaffo, 2020). It occurs more frequently in males than in females and significantly lowers life quality while imposing a heavy economic burden (Danve and Neogi, 2020). Current treatments include nonsteroidal anti-inflammatory drugs, colchicine, and corticosteroids, which may cause side effects such as hepatorenal toxicity and cardiovascular issues (Zeng et al., 2023). Moreover, gout commonly leads to acute joint pain and is linked with systemic diseases such as hypertension (Khanna et al., 2020), type 2 diabetes (Borghi et al., 2020), and cardiovascular diseases (Cox et al., 2021). Numerous studies have identified genetic variants linked to gout, but the biological implications of these findings are unclear (Dehghan et al., 2008; Li et al., 2015; Nakayama et al., 2017; Chen et al., 2018), These variants, identified through GWAS, likely affect the disease through gene expression, suggesting that exploring this relationship can clarify the regulatory pathways in gout’s pathogenesis.

Mendelian Randomization (MR) is a method that uses genetic variants to examine potential causal relationships between exposures and outcomes, thus reducing confounding factors. A novel analytical framework that integrates SMR with cis-eQTL and GWAS data has been proposed (Nica and Dermitzakis, 2013; Ni et al., 2020). This method has identified genes with pleiotropic or potential causal associations with various phenotypes, such as polycystic ovary syndrome (Sun et al., 2022), nasal polyp (Yoshikawa et al., 2023), and periodontitis (Wang et al., 2021), demonstrating its value in investigating disease-related gene pleiotropy.

In this study, we applied the SMR approach to integrate GWAS and cis-eQTL data for gout, prioritizing genes that may have pleiotropic or potential causal relationships with the disease.

## 2 Materials and methods

### 2.1 Data sources

The cis-eQTL data were sourced from two primary datasets. Firstly, summary-level data from the eQTLGen Consortium (Võsa et al., 2018), which includes 37 datasets and 31,684 participants, is available at https://www.eqtlgen.org/cis-eqtls.html. Secondly, data from the V8 release of the GTEx eQTL summarized data for kidney (GTEx Consortium, 2020) tissue, involving 73 participants, can be downloaded at https://yanglab.westlake.edu.cn/software/smr/#eQTLsummarydata. The GWAS data for gout was sourced from the FinnGen Documentation of the R10 release (Kurki et al., 2023), comprising 9,568 gout cases and 262,844 controls. This data is available for download at https://finngen.gitbook.io/documentation/data-download. All summary data in this study are publicly accessible and have obtained ethical approval from the respective institutions (Table 1).

**TABLE 1:** Details of the cis-eQTL and GWASs included in the summary data-based Mendelian randomization.

Trait	Data type	Total number of participants	Consortium
Blood	Exposure	31,684	eQTLGen
Kidney	Exposure	73	GTEx_V8
Gout	Outcome	272,412	FinnGen_R10

### 2.2 SMR analysis

**FIGURE 1:**
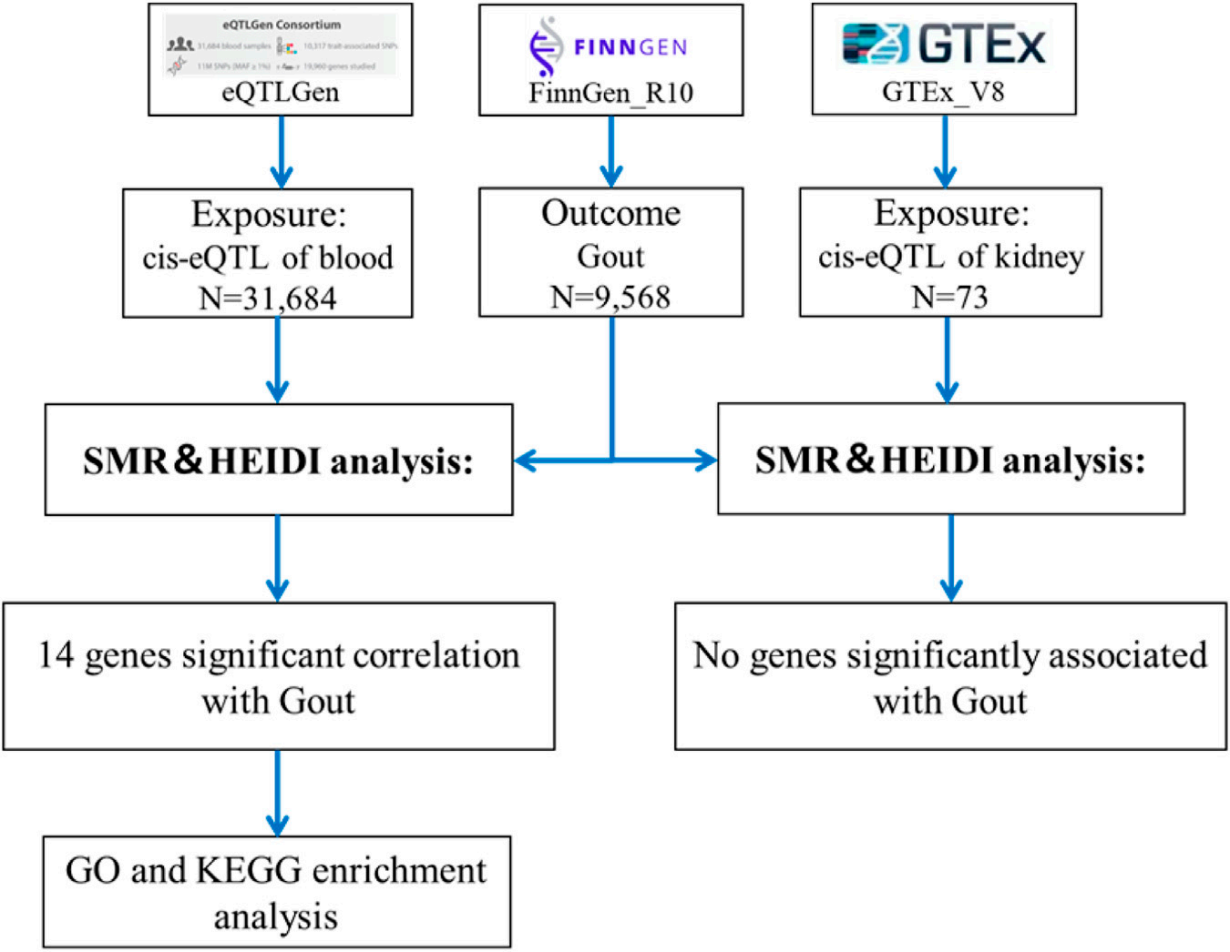
Flowchart of SMR analysis.

The SMR analysis utilized summary statistics from eQTL and GWAS datasets to examine the association between gene expression and gout. This analysis employed SNP markers from cis-eQTLs as instrumental variables (IVs), with gene expression as the exposure and gout as the outcome. The IVs must satisfy three critical criteria for MR validity: 1) IVs must be strongly associated with gene expression; 2) IVs should not be related to any confounders; 3) IVs must influence the outcome exclusively through gene expression and not through other pathways (Yang et al., 2021). The analysis was conducted using SMR software version 1.3.1, following default settings, including pruning SNPs with a Minor Allele Frequency (MAF) greater than 0.01, selecting cis-eQTL at p < 5 × 10^−8^, excluding SNPs with linkage disequilibrium (LD) r-squared between top-SNP greater than 0.90 or less than 0.05, and eliminating one SNP of each pair with LD r-squared greater than 0.90 (Zhu et al., 2016). Significant gene loci were identified with a P_SMR less than 0.05. Heterogeneity tests (HEIDI) were conducted to assess the robustness of the associations (P_HEIDI greater than 0.05), and results were adjusted using the False Discovery Rate (FDR) method (FDR <0.05) (Zhang Q. et al., 2023). The SMR analysis process is depicted in Figure 1.

### 2.3 Gene ontology (GO) and KEGG analyses

To explore potential biological functions and pathways, analyses were performed using RStudio version 4.3.2. Enrichment analysis was conducted using the clusterProfiler package and the enrichGO function (Rosado-Galindo and Domenech, 2023; Zheng et al., 2024), with the gene ontology database set to org.Hs.eg.db (Wang et al., 2022). The resulting GO diagrams illustrate the biological processes, molecular functions, and cellular components significantly related to differentially expressed genes.

## 3 Results

**FIGURE 2:**
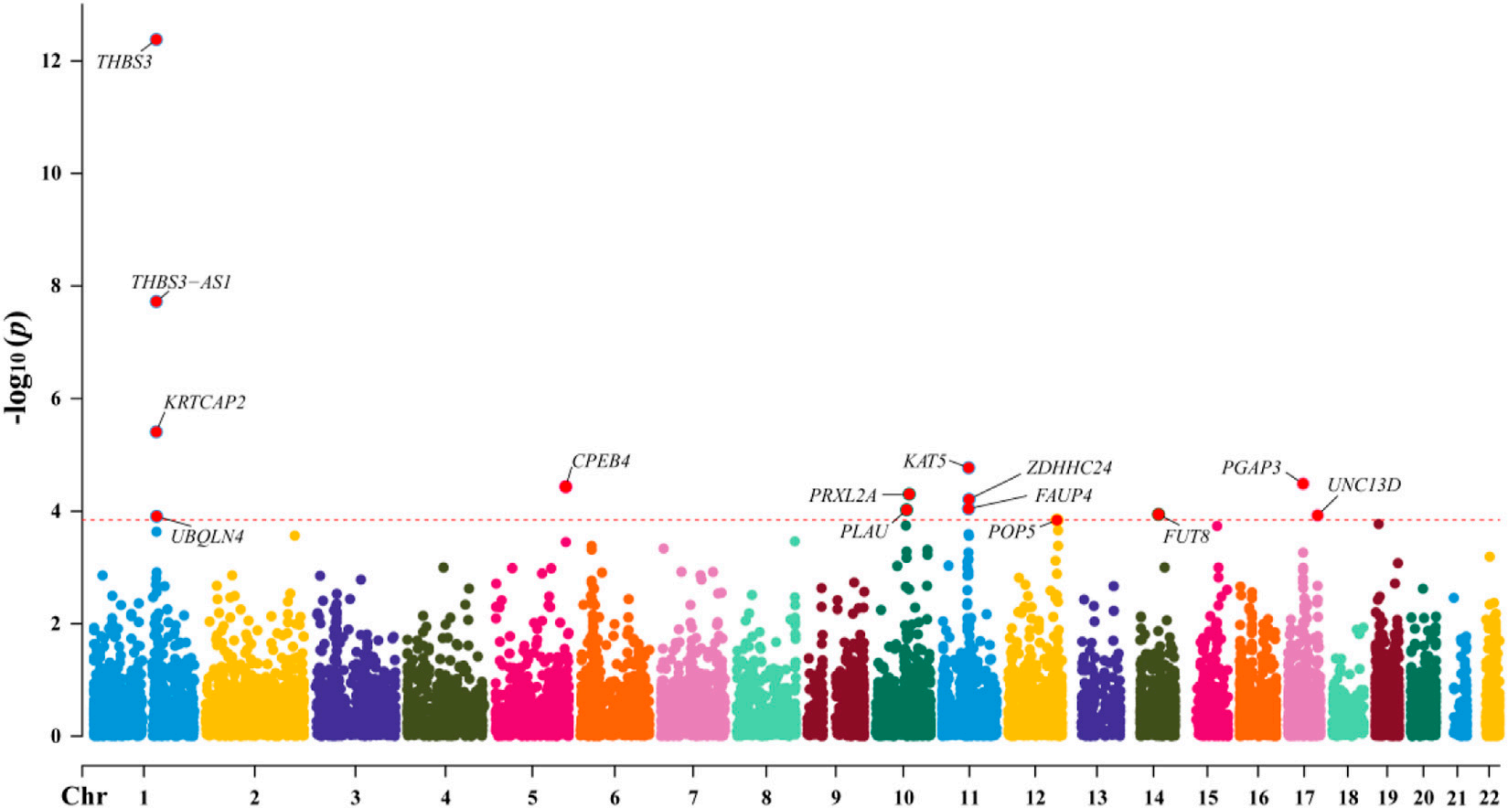
Manhattan Plot of SMR Analysis Results between eQTL and Gout.

Through SMR analysis, and subsequent filtering using P_SMR, P_FDR, and P_HEIDI values, 15,679 probes in the eQTLGen blood data were found to be associated with gout. Among these, 14 genes showed pleiotropic or potential causal relationships with gout. No significant gene associations with gout were detected in the GTEx V8 kidney tissue data, attributed to the limited sample size of kidney tissue. Details regarding these genes are presented in Figure 2 and Table 2.

**TABLE 2:** Genes significantly associated with Gout in SMR analysis.

Ensemble ID	CHR	Gene	Top SNP	SMR		HEIDI	FDR
				Beta	P-Value	P-Value	P-Value
ENSG00000169231	1	THBS3	rs760077	−0.202	4.16 × 10^−13^	0.219	2.92 × 10^−9^
ENSG00000231064	1	THBS3-AS1	rs760077	−1.162	1.88 × 10^−8^	0.882	3.30 × 10^−5^
ENSG00000163463	1	KRTCAP2	rs12752585	1.306	3.88 × 10^−6^	0.118	3.03 × 10^−3^
ENSG00000172977	11	KAT5	rs502468	−1.508	1.70 × 10^−5^	0.304	1.11 × 10^−2^
ENSG00000161395	17	PGAP3	rs2952152	0.191	3.24 × 10^−5^	0.947	1.89 × 10^−2^
ENSG00000113742	5	CPEB4	rs72810995	−0.095	3.66 × 10^−5^	0.853	2.06 × 10^−2^
ENSG00000122378	10	PRXL2A	rs10788630	−0.172	4.96 × 10^−5^	0.093	2.49 × 10^−2^
ENSG00000174165	11	ZDHHC24	rs3737525	0.536	6.15 × 10^−5^	0.227	2.87 × 10^−2^
ENSG00000173727	11	FAUP4	rs1621277	−0.252	9.05 × 10^−5^	0.118	3.63 × 10^−2^
ENSG00000122861	10	PLAU	rs2227551	0.262	9.44 × 10^−5^	0.077	3.68 × 10^−2^
ENSG00000033170	14	FUT8	rs8010726	−0.187	1.14 × 10^−4^	0.451	4.09 × 10^−2^
ENSG00000092929	17	UNC13D	rs2290769	−0.318	1.19 × 10^−4^	0.425	4.16 × 10^−2^
ENSG00000160803	1	UBQLN4	rs34444588	−0.242	1.23 × 10^−4^	0.144	4.22 × 10^−2^
ENSG00000167272	12	POP5	rs492574	−0.228	1.44 × 10^−4^	0.878	4.81 × 10^−2^

**FIGURE 3:**
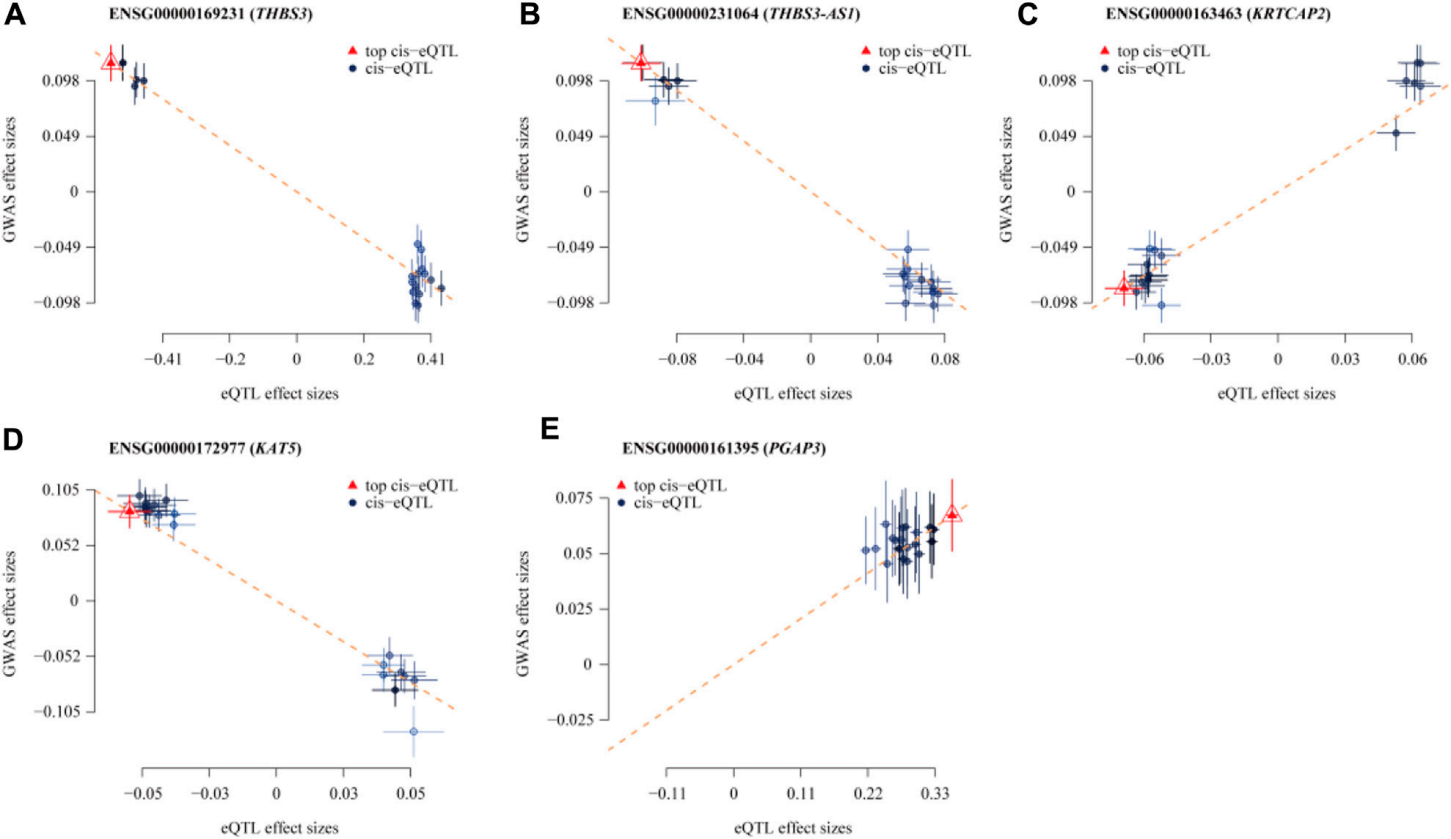
Scatterplot of the Association Results between Individual Genes in eQTL and Gout. **(A)** THBS3; **(B)** THBS3-AS1; **(C)** KRTCAP2; **(D)** KAT5; **(E)** PGAP3. The x-axis shows the effect sizes of SNPs within the eQTL genes, and the y-axis shows the effect sizes of SNPs associated with gout. Blue circles indicate SNPs, and red triangles mark the top SNPs. The dashed lines show the regression line, highlighting the direction of the association between eQTL effect sizes and GWAS effect sizes. Segments ascending from left to right indicate a positive correlation between gene expression and disease, implying that higher gene expression is linked to an increased risk of gout. Conversely, decreasing segments suggest a negative correlation, where higher gene expression is linked to a reduced risk of gout.

**FIGURE 4:**
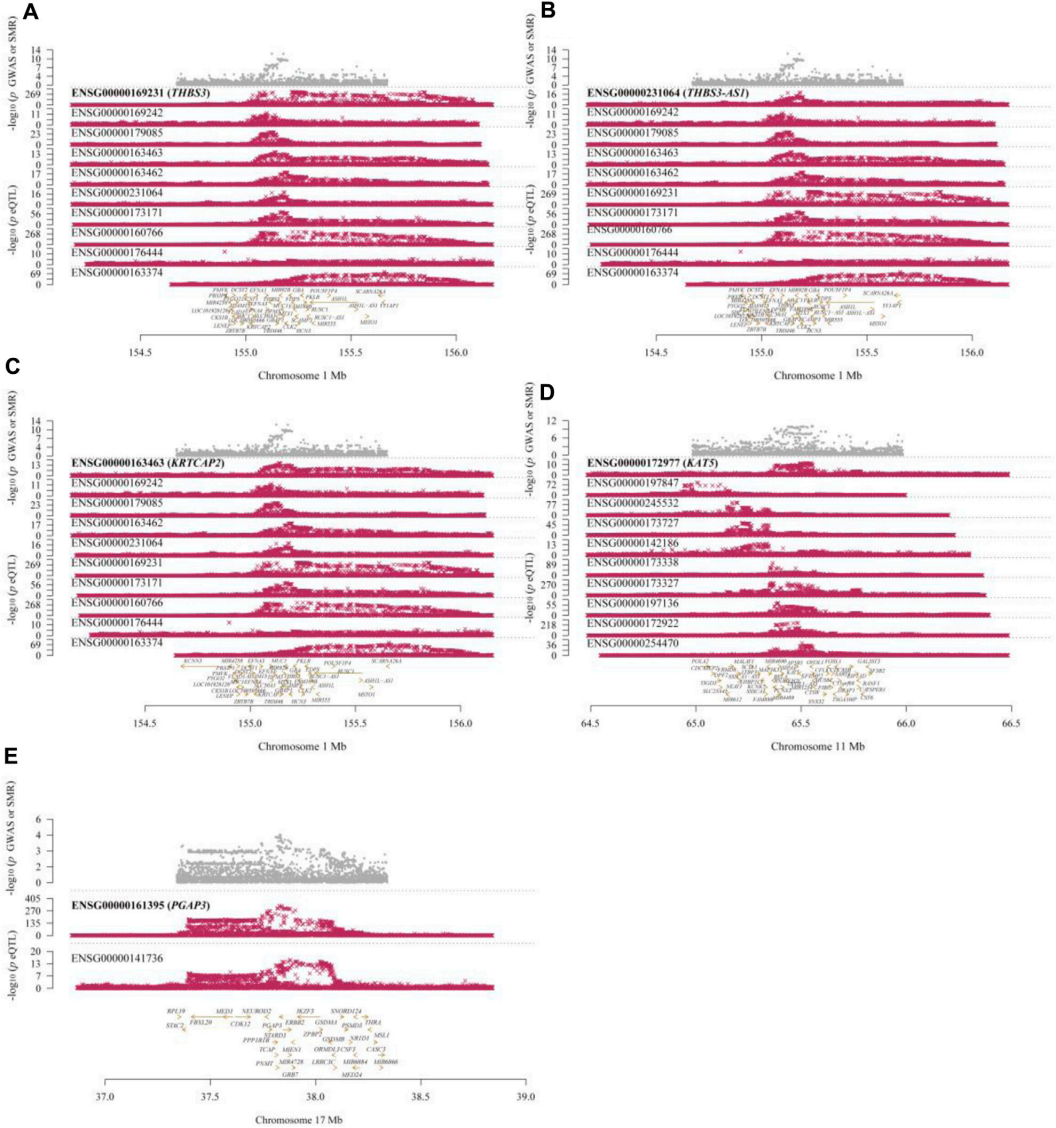
Locus Plots Showing the Pleiotropic Associations between Significant Genes and Gout. **(A)** THBS3; **(B)** THBS3-AS1; **(C)** KRTCAP2; **(D)** KAT5; **(E)** PGAP3. The top gray dots represent the -log10(*p*-values) of SNPs from GWAS. The middle red dots indicate the -log10(*p*-values) of the genes, and the bottom section displays the positions of the probes relative to the genes.

In the eQTLGen dataset, the top five genes identified include ENSG00000169231 (labeled THBS3, P_SMR = 4.16 × 10^−13^), ENSG00000231064 (labeled THBS3-AS1, P_SMR = 1.88 × 10^−8^), ENSG00000163463 (labeled KRTCAP2, P_SMR = 3.88 × 10^−6^), ENSG00000172977 (labeled KAT5, P_SMR = 1.70 × 10^−5^), and ENSG00000161395 (labeled PGAP3, P_SMR = 3.24 × 10^−5^). Elevated expression of KRTCAP2 and PGAP3 is linked to an increased risk of gout, whereas higher expression of THBS3, THBS3-AS1, and KAT5 is associated with a reduced risk of the disease. Data from these five genes were specifically extracted and displayed on scatter plots and locus plots to depict the associations between these genes and the disease (Figures 3, 4).

**FIGURE 5:**
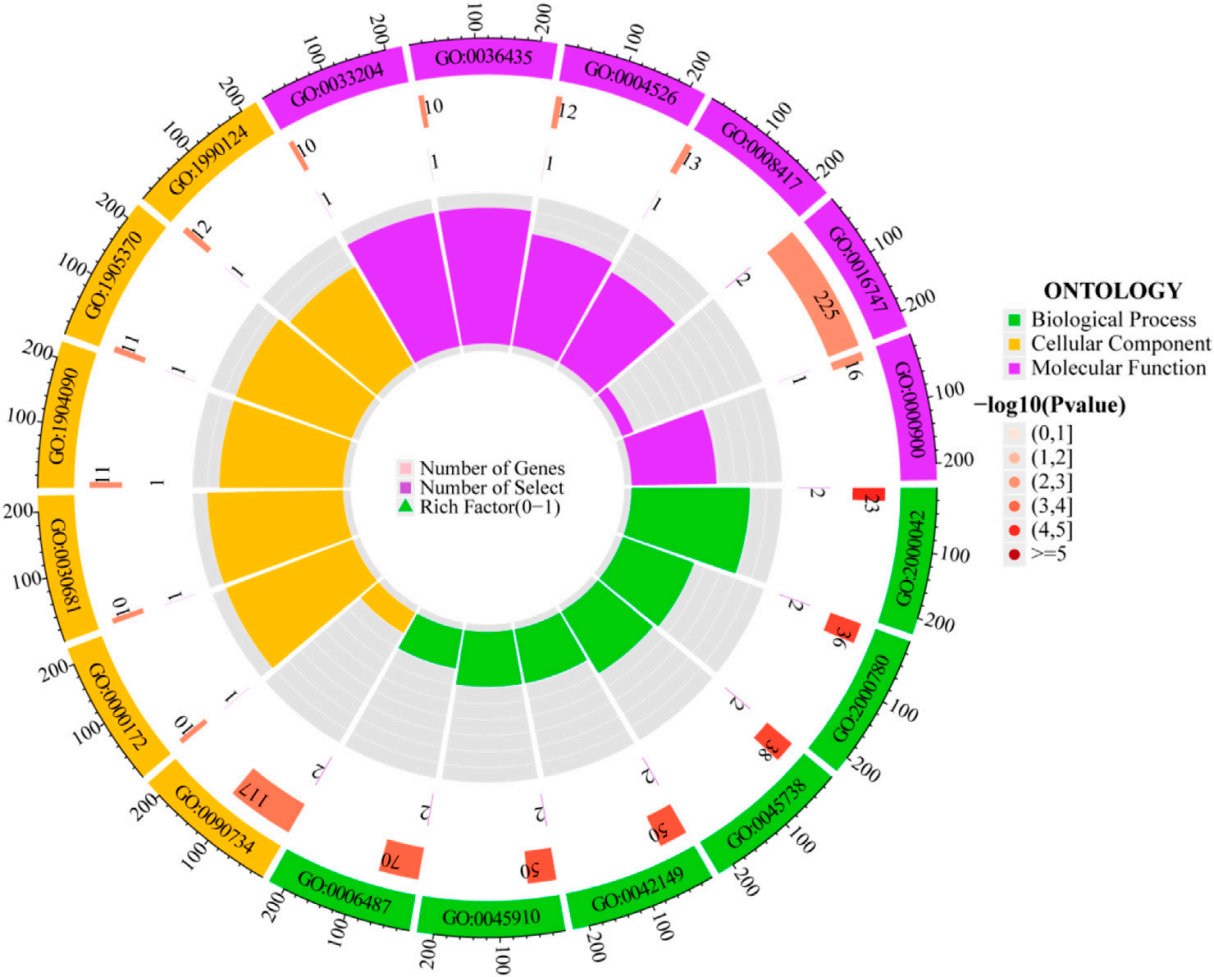
Circular diagram of GO enrichment analysis.

Through GO enrichment analysis, identifications were made of 182 biological processes, 23 cellular components, and 21 molecular functions. The top six biological processes include: negative regulation of double-strand break repair via homologous recombination, negative regulation of double-strand break repair, negative regulation of DNA repair, cellular response to glucose starvation, negative regulation of DNA recombination, and protein N-linked glycosylation. The top six cellular components are: the site of DNA damage, ribonuclease MRP complex, multimeric ribonuclease P complex, peptidase inhibitor complex, serine-type endopeptidase complex, and messenger ribonuclease P complex. The top six molecular functions comprise: ribonuclease P RNA binding, K48-linked polyubiquitin modification-dependent protein binding, ribonuclease P activity, fucosyltransferase activity, acyltransferase activity transferring groups other than amino-acyl, and mRNA regulatory element binding translation repressor activity. The visualization of the top-ranked biological processes from the three categories of GO enrichment analysis is shown in Figure 5. Additionally, KEGG pathway analysis identified five pathways: Transcriptional Misregulation in Cancer; Glycosaminoglycan Biosynthesis - Keratan Sulfate; Various Types of N-Glycan Biosynthesis; Malaria; and N-Glycan Biosynthesis, primarily related to glycan biosynthesis and metabolism. Detailed GO and KEGG information is available in Supplementary Tables S1, S2; Supplementary Figures S1, S2.

The outermost ring displays the GO enrichment IDs, the second ring shows the number of genes, the third ring depicts the number of genes significantly associated with the disease, and the fourth ring illustrates the proportion of significant genes. Three different colors represent various biological processes. The color intensity in the second ring indicates the -log10 (p-value) of gene enrichment, the deeper the red, the more significant the enrichment of disease-related genes.

## 4 Discussion

This study merged gout-related GWAS and cis-eQTL data for SMR analysis to identify genes with pleiotropic or potential causal relationships to gout. We identified 14 genes implicated in the pathogenesis of gout, focusing on the top five genes discussed herein.

In our findings, THBS3 and THBS3-AS1 exhibited significant pleiotropic associations with a reduced risk of gout. They are also linked to clear cell renal cell carcinoma (Wang et al., 2023), osteoarthritis (Costa et al., 2024), and stomach cancer (Deng et al., 2021). THBS3, a pentameric protein of the extracellular matrix (ECM) family, includes over 12 kb, with 3.1 kb of exon sequences and 9.2 kb of introns (Adolph et al., 1995). As a member of the thrombospondin family, it supports various biological processes such as ECM interactions, cell adhesion, and inflammatory responses (Chen et al., 2022; Yang et al., 2023), potentially affecting immune cell infiltration and activity at inflammation sites, thereby influencing the progression of gout. A genetic epidemiological study provided evidence for the critical role of variations in the TRIM46-MUC1-THBS3-MTX1 gene region in the pathogenesis of kidney and blood diseases, underscoring their importance in hyperuricemia and gout development (Teng et al., 2021). Furthermore, THBS3-AS1, a long non-coding RNA (lncRNA), is crucial in gene expression regulation, cellular processes, and pathogenesis. The relationship between THBS3-AS1 and gout remains less explored, and we hypothesize that it could influence gout through modulation of inflammatory cell activity or by regulating the expression of urate handling genes, warranting further research.

Additionally, KRTCAP2 was identified as a primary gene increasing the risk of gout. Changes in protein glycosylation can impact immune responses, with KRTCAP2 situated on human chromosome 1q22, encoding a protein involved in glycosylation, which plays a crucial role in biological functions like cell recognition, immune response, and signal transduction (Sun et al., 2023). Literature suggests that KRTCAP2 may affect urate production and clearance by altering the expression and function of xanthine oxidoreductase (Lee et al., 2022). However, the regulation of XOR gene expression by KRTCAP2 depends primarily on the modulation of core transcription factors such as Sp1 or PPARγ (DeVallance et al., 2023).

KAT5, from the MYST acetyltransferase family, significantly influences various cellular activities and was associated with a reduced risk of gout in our study. It modulates chronic inflammatory responses in different forms of arthritis by regulating Foxp3 expression in regulatory T cells and STAT6 in B cells (Yang et al., 2018; Su et al., 2019). Earlier research indicates that a loss or reduction of KAT5 function may alleviate systemic inflammatory responses in MSU-induced peritonitis models, commonly linked with gout or urate deposition (Zhang Y. et al., 2023).

Furthermore, PGAP3 increases the risk of gout. This protein participates in the modification of glycosylphosphatidylinositol (GPI) anchors, a key step in a major metabolic pathway for hyperuricemia (Yang et al., 2019). In GPI anchor biosynthesis, PGAP3’s essential role is to refine newly synthesized GPI anchors by eliminating non-native acyl groups, essential for the proper expression and functionality of these anchors (Howard et al., 2014). Increased expression of PGAP3 alters GPI anchor modifications, thereby influencing urate metabolism in the body.

This study has several limitations. Firstly, the limited number of probes in our SMR analysis and the small sample size in the eQTL analysis might have led to the omission of crucial genes associated with gout. Future studies are advised to utilize larger samples in eQTL analysis to identify more genes contributing to the pathogenesis of gout. Secondly, this study included only participants of European ancestry. Additional research is needed to apply these findings to other ethnic groups. Thirdly, the small sample size of only 73 participants in the GTEx V8 kidney tissue data may have prevented the identification of significant gene associations, resulting in inadequate statistical power. Future research should use larger sample sizes for kidney tissue data to increase statistical power and improve the reliability of the results. Fourthly, the relevance of the HEIDI test for some identified genes implies that horizontal pleiotropy cannot be discounted, suggesting that the observed associations could be due to the effects of two distinct genetic variants in strong linkage disequilibrium.

## 5 Conclusion

In summary, this study combined GWAS and eQTL data related to gout, identifying 14 genes that may be involved in the pathogenesis of the disease. These genes are implicated in regulating inflammatory responses, immune reactions, and uric acid metabolism. However, further research is necessary to confirm the functions of these genes in gout and to investigate other genes related to the disease’s mechanisms.

## Data Availability

The original contributions presented in the study are included in the article/Supplementary Material, further inquiries can be directed to the corresponding author/s.
