# Supplementary material for: Mendelian randomization analysis identified potential genes pleiotropically associated with gout
Source: Front Genet. 2024 Aug 5;15:1426860. doi: 10.3389/fgene.2024.1426860 (PMC11330811; doi:10.3389/fgene.2024.1426860)

Supplementary Material

**Supplementary Figure S1.**

Barplot of KEGG enrichment analysis of genes significantly associated with Gout.


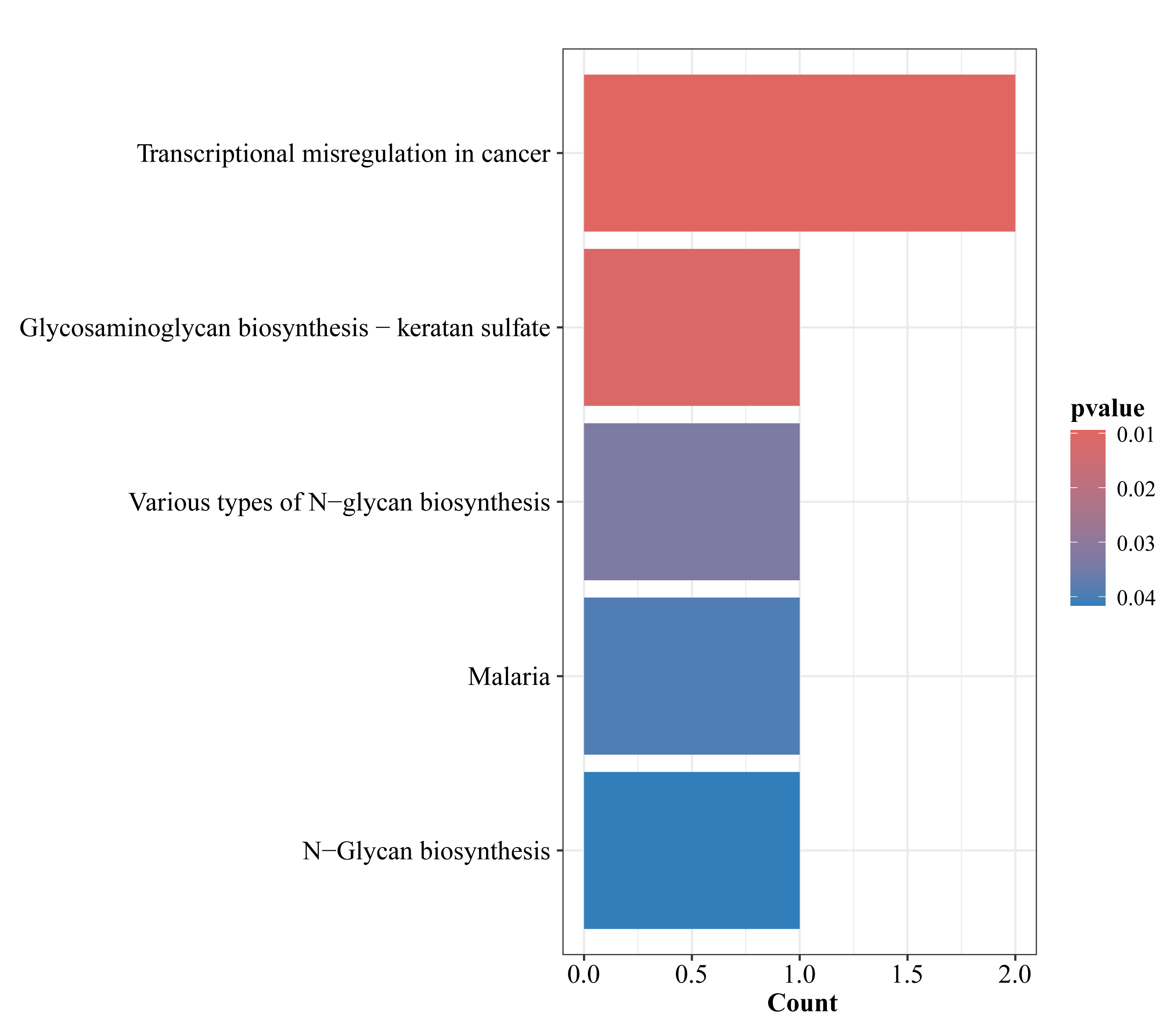


**Supplementary Figure S2.**

Barplot of GO enrichment analysis of genes significantly associated with Gout.


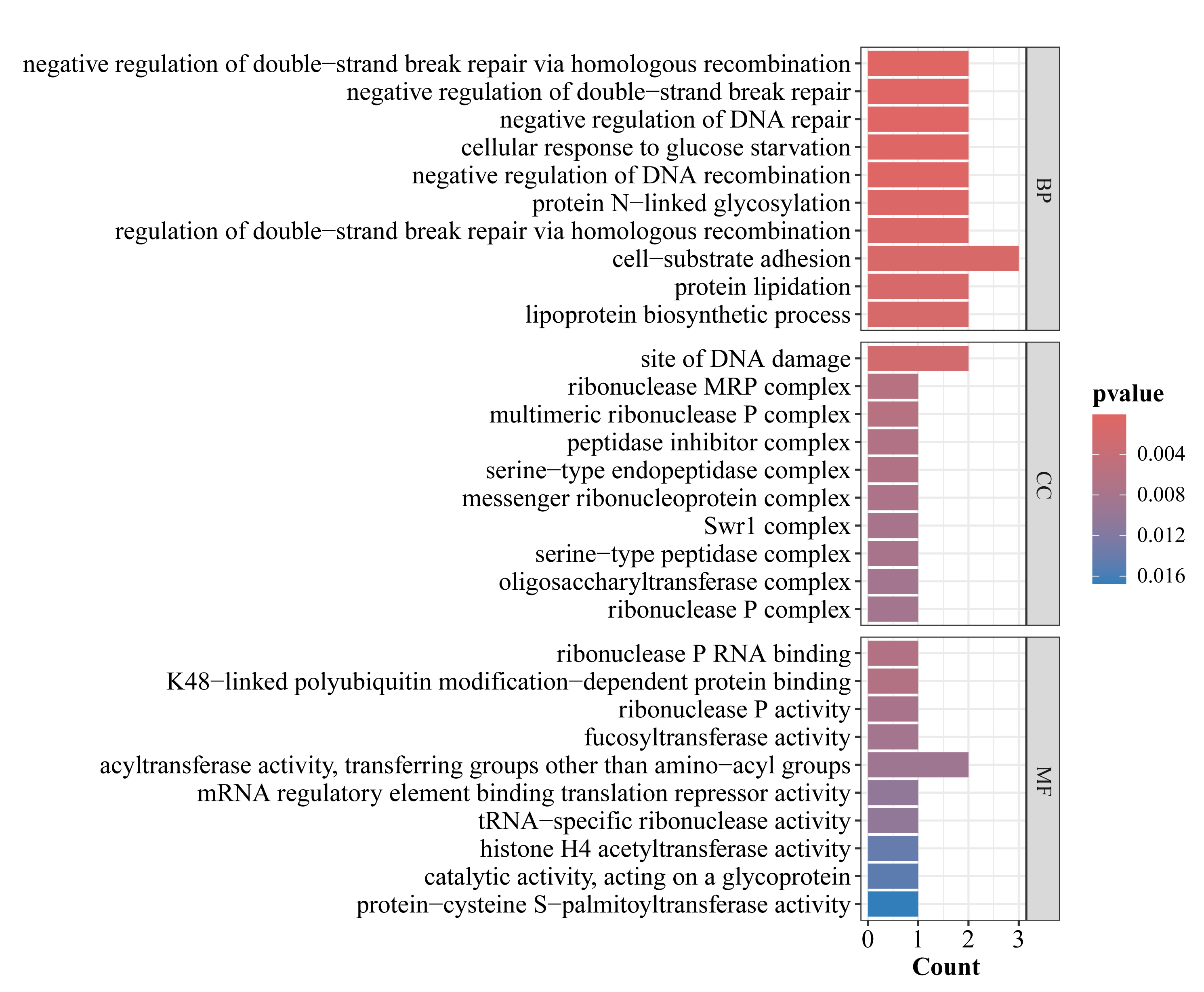

Supplement: Supplementary file 2 [file Table1.DOCX]
